# Three Years None Too Long for a Dental Course

**Published:** 1899-12

**Authors:** George Brooks

**Affiliations:** Greenfield, Iowa


### THREE YEARS NONE TOO LONG FOR A DENTAL COURSE.

BY GEORGE BROOKS, D.D.S., GREENFIELD, IOWA.

In the August number of the INTERNATIONAL DENTAL JOURNAL appeared a paper entitled "Are we punishing the Right Man?" in which the writer holds that twenty-one months is longer than is needed for some men to fit themselves for the practice of dentistry, and that they are simply held back by the slow ones who do need that time. The method suggested by the writer would, perhaps, be better than the present way, but it would incur many complications, and I think it would not obtain the best ends, nor raise the profession to nearly so high a plane as the plan suggested by Waldo E. Royce, D.D.S., of Tunbridge Wells, England, in his article, "Some Suggestions in regard to Dental Examinations in the United States," which appeared in the June number of the JOURNAL. His plan, you will remember, was to create a national board and a national diploma, which it would require the best of training to obtain, and which would be recognized as genuine in any nation on the globe and the best that could be obtained.

In the Freshman dental class of the State University of Iowa, in the fall of 1895, there were one hundred and twenty-seven members, of which, in 1898, only sixty-two graduated, showing a decrease of sixty-five, over one-half. Of the sixty-five who failed to graduate, perhaps one-half sought other schools because of the reports of large clinics and the great amount of practical work to be done, of which they had but little at the State University of Iowa in the first year. The other half of the sixty-five left because they failed in two or more studies, and sought easier schools or decided that they had chosen the wrong profession. Several of the sixty-two who stayed were back in two, and a few in three, studies until the last year. The fact was, that every man in the class had an opportunity to study all he wished, and I think there was not a man in the class who did not feel, as he graduated, that he needed more of both theory and practice.

What I wish to show is, that the best men had all the work that they wanted to do, and many of the one hundred and twenty-seven had much more than they could do. The very best did not pass perfect examinations, and the poorest barely got through. I think it would be a rare exception for a student to do this work in nine months (who had not had previous work in this line), as the doctor tells us is accomplished by the University of Virginia. There is not a day goes by but the ability of a dentist is questioned, and I suppose there is seldom a month goes by but he makes some mistakes in his work that will admit of question. We all make failures, and our course at school is none too long to fit us for our work.

There are many first-class men who have to compete with the man who advertises, and it is a question with him how he is to gain a practice and yet not step over the lines of dental ethics. The national diploma would help him out of his difficulties, because the letter "N" added to his degree (when once understood by the people) would be worth more than all the advertising one could do. It is a part of Dr. Royce's plan that any man forfeits his degree and diploma if he advertises or indulges in other unprofessional practices.

If any State board would make the national diploma necessary to the practice of dentistry in that State, the unworthy and unprofessional dentist would have to seek a new field. Much would depend on the National board. This examination should be as strict as, if not stricter than, that given by any dental college or State board in the Union, and I think one of the requirements should be that a candidate for a national diploma shall have had at least one year's practice, after graduating, before he could secure the degree. I earnestly hope to see the national degree a reality, soon, and that it may not become valueless on account of the ease with which it may be obtained, but that it will be worthy of the effort of the best men and be enhanced in value by the work of the men who hold them.

